# Evaluation of sublingual microcirculation in a paediatric intensive care unit: prospective observational study about its feasibility and utility

**DOI:** 10.1186/s12887-017-0837-5

**Published:** 2017-03-15

**Authors:** Rafael González, Jorge López, Javier Urbano, María José Solana, Sarah Nicole Fernández, María José Santiago, Jesús López-Herce

**Affiliations:** 1 Paediatric Intensive Care Unit, Gregorio Marañón General University Hospital, Calle Doctor Castelo 47, Madrid, 28009 Spain; 2 Gregorio Marañón Health Research Institute, Calle Doctor Castelo 47, Madrid, 28009 Spain; 3 Mother and Child Health and Development Network (Red SAMID), RETICS funded by the PN I+D+I 2008-2011, ISCIII-Sub-Directorate General for Research Assessment and Promotion and the European Regional 10.1186/s12887-017-0837-5 Development Fund, ref. RD12/0026., Madrid, Spain; 4 School of Medicine, Complutense University of Madrid, Madrid, Spain

**Keywords:** Microcirculation, Paediatric intensive care, Shock, Monitoring, Critically ill child

## Abstract

**Background:**

Evaluation of the microcirculation in critically ill patients is usually done by means of indirect parameters. The aim of our study was to evaluate the functional state of the microcirculation by direct visualization of sublingual microcirculation using Sidestream Dark Field Imaging, to determine the correlation between these findings and other parameters that are commonly used in the clinical practice and to assess the applicability of the systematic use of this technique in critically ill children.

**Methods:**

A prospective observational study was carried out in a Pediatric Intensive Care Unit (PICU) of a tertiary referral hospital. All patients admitted to the PICU during a three-month period were included in the study after obtaining the informed consent from the patient. Systematic evaluation of sublingual microcirculation was done in these patients (Total Vessel Density, Proportion of Perfused Vessels, Perfused Vessel Density, De Backer Score, Microvascular Flow Index, Heterogeneity Index) within the first day of admission (T_1_) and between the second and third day of admission (T_2_). Other clinical, hemodynamic, and biochemical parameters were measured and registered simultaneously. When the evaluation of the microcirculation was not feasible, the reason was registered. Descriptive analysis of our findings are expressed as means, medians, standard deviations and interquartile ranges. Mann–Whitney-Wilcoxon and Fisher tests were used to compare variables between patients with and without evaluation of the microcirculation. Pearson Correlation Coefficient (ρ) was used to evaluate the correlation between microcirculatory parameters and other clinical parameters.

**Results:**

One hundred fine patients were included during the study period. Evaluation of the microcirculation was feasible in 18 patients (17.1%). 95.2% of them were intubated. The main reason for not evaluating microcirculation was the presence of respiratory difficulty or the absence of collaboration (95.1% on T_1_ and 68.9% on T_2_). Evaluated patients had a higher prevalence of intubation and ECMO at admission (72.2% vs. 14.9% and 16.6% vs. 1.1%, respectively), and longer median duration of mechanical ventilation (0 vs. 6.5 days), vasoactive drugs (0 vs. 3.5 days) and length of stay (3 vs. 16.5 days) than non-evaluated patients. There was a moderate correlation between microcirculatory parameters and systolic arterial pressure, central venous pressure, serum lactate and other biochemical parameters used for motoring critically ill children.

**Conclusions:**

Systematic evaluation of microcirculation in critically ill children is not feasible in the unstable critically ill patient, but it is feasible in stable critically ill children. Microcirculatory parameters show a moderate correlation with other parameters that are usually monitored in critically ill children.

**Electronic supplementary material:**

The online version of this article (doi:10.1186/s12887-017-0837-5) contains supplementary material, which is available to authorized users.

## Background

The role of the hemodynamic system is to guarantee an appropriate delivery of oxygen and nutrients to the different organs and tissues. Two compartments can be distinguished in the hemodynamic system: the macrovascular and the microvascular compartment. The microvascular compartment is responsible for the final delivery of oxygen and nutrients to the tissues through convection and diffusion mechanisms [1].

Clinical evaluation of microcirculation is usually done by means of indirect and subjective estimations such as skin coloration and capillary refill time [2].

Some new techniques for direct visualization of the microvascular compartment have been developed in the last few years, such as sidestream dark field (SDF) microscopy [3] and orthogonal polarization spectral (OPS) imaging [4, 5].

Some studies show that direct visualization of the microcirculation has prognostic implications and can be useful for guiding further management of the critically ill patient [6–13]. Nevertheless, these techniques are not yet widely used in routine clinical practice.

Several studies have analysed the use of SDF in children with haemorrhagic or septic shock [7, 11, 14–19], but its usefulness as a way to evaluate microcirculation in critically ill children regardless of their underlying condition or clinical situation has not been evaluated. Nor are there any studies providing a definition of what is normal for the different microcirculatory parameters or what the prevalence of microcirculatory alterations are in children.

Another important factor is that most of these monitoring techniques have been designed for adult patients, and their use in children is more complicated due to differences in size, physiology and lack of cooperation.

### Aim of the study

The main objective of this study was to analyse the applicability of the systematic evaluation of sublingual microcirculation using SDF in critically ill children. Secondary objectives were to study the prevalence of microcirculatory alterations, the correlation with other hemodynamic parameters used in routine clinical practice and to analyse the prognostic power of the different microcirculatory parameters.

## Methods

### Study design, patients and study site

A prospective observational study was conducted. The study was approved by the local Clinical Research Ethics Committee. Patients that had been admitted for more than 24 h in the Paediatric Intensive Care Unit (PICU) were included during a three month period (between 15 October 2013 and 15 January 2014). Patients or their legal representatives were asked for written informed consent to participate in the study. The patients that did not give written informed consent for participating in the study were excluded.

The study took place in the PICU of a tertiary referral hospital which counts with 11 beds and paediatric surgery, paediatric cardiac surgery and extracorporeal membrane oxygenation (ECMO) programs. Between the years 2008 and 2013 the Unit had a mean of 438.8 ± 166.3 admissions per year and a mean length of stay of 7.8 ± 2.8 days. The age of the patients admitted to the PICU ranged between 1 month and 18 years.

### Variables

The following variables were registered for each patient during the study period: date of birth, size and weight, reason for admission and whether it was programmed or not, intubation at admission, mechanical ventilation (MV) parameters, duration of MV, need for and duration of vasoactive drug therapy, renal replacement therapies or ECMO, PICU length of stay and mortality.

Sublingual microcirculation was evaluated within the first day of admission (T_1_) and between the second and third days of admission (T_2_). The following variables were registered simultaneously: heart rate (HR), systolic arterial pressure (SAP), diastolic and mean arterial pressures (DAP and MAP, respectively), central venous pressure (CVP), transcutaneous oxygen saturation (SatO_2_%), core and peripheral body temperature, tissue oxygen saturation (measured by Near Infra-Red Spectrophotometry (NIRS)); analytical values: arterial and venous pH, pO2, pCO2, HCO3, oxygen saturation, serum lactate; haemoglobin concentration, leukocyte and platelet count; treatment (vasoactive drugs, vasoactive-inotropic score [20] (dose of dopamine (μg/kg/min) + dose of dobutamine (μg/kg/min) + 100 × dose of epinephrine (μg/kg/min) + 10 × dose of milrinone (μg/kg/min) + 10000 × dose of vasopressin (U/kg/min) + 100 × dose of norepinephrine (μg/kg/min)), respiratory support: peak inspiratory pressure, mean airway pressure and fraction of inspired oxygen (FiO2).

Reasons for the failure to evaluate sublingual microcirculation were registered for each patient, and if it was due to clinical reasons (lack of cooperation, the presence of respiratory distress that may worsen with the procedure, etc.) or to the lack of qualified and trained personnel to carry out the evaluation.

### Evaluation of sublingual microcirculation

**Fig. 1:**
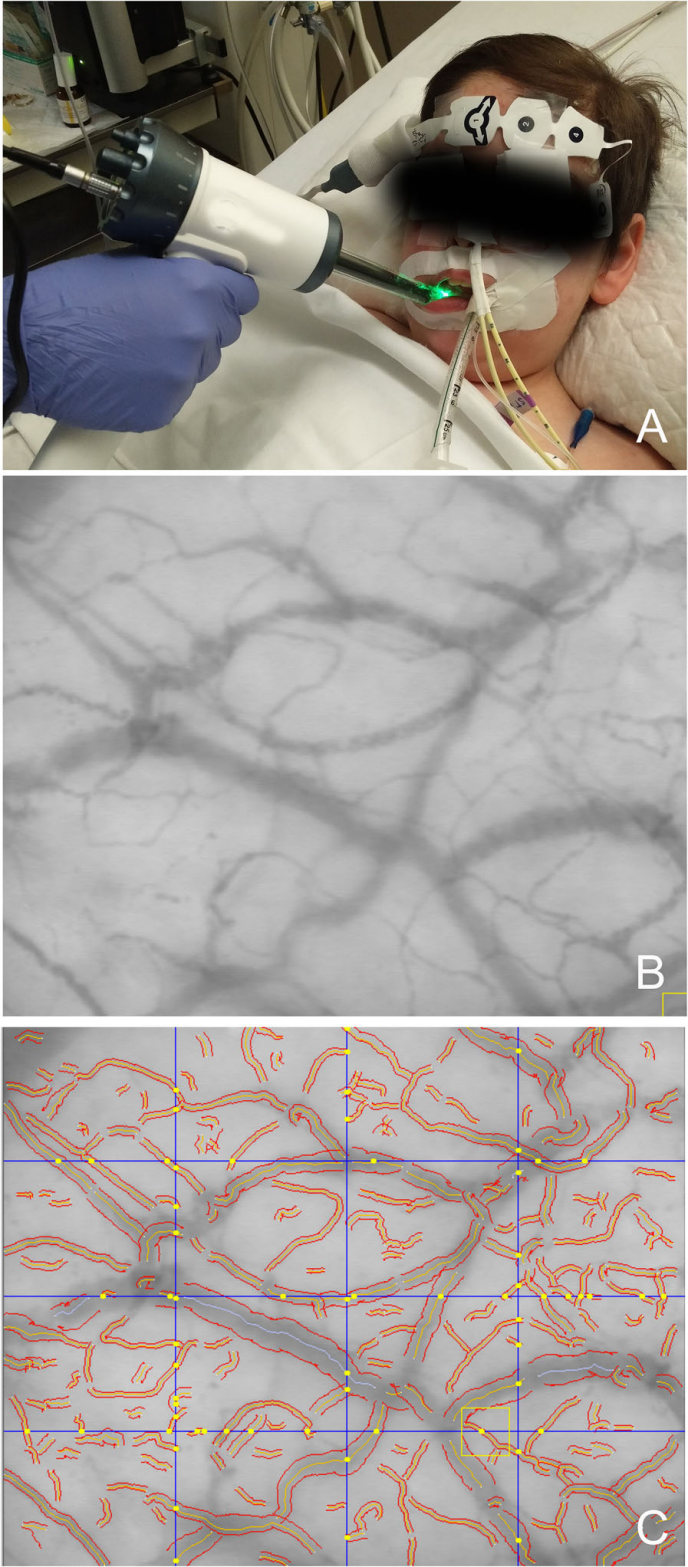
Evaluation of sublingual microcirculation using SDF imaging device. **a** Device is applied to the patient on the sublingual area. **b** Microcirculatory image acquired by the device. **c** Vessels are identified during analysis (in red) allowing calculation of microcirculatory parameters. Crossing points (in yellow) with three equidistant vertical and horizontal lines are marked to calculate De Backer Score

International recommendations for imaging acquisition and analysis of sublingual microcirculation were followed [21]. Microscan® (Microvision Medical, Amsterdam, The Netherlands) device was used for SDF video image acquisition. After gentle secretion removal, images from both sides of the sublingual region were captured (5 video sequences of 20 s each) for both evaluation periods (T_1_ and T_2_). Figure 1 shows how video sequences are acquired and how vessels are identified in each video sequence to evaluate microcirculatory parameters.

Captured sequences were then analysed by a single investigator that was blind to the moment of the evaluation and to the identity and clinical condition of the patient. Video sequences with artifacts (due to excessive pressure, inadequate focusing or instability) were removed from the analysis.

Automated Vascular Analysis software AVA ® Software 3.0 (Microvision Medical, Amsterdam, The Netherlands) was used for analysis. Several parameters were analysed in each sequence: Microvascular Flow Index (MFI), Total Vascular density (TVD), Perfused Vessel density (PVD), De Backer score [22] and Proportion of Perfused Vessels (PPV%). Heterogeneity index (HI) was also calculated for each set of measurements.

MFI evaluates the predominant flow type in the examined site of each video sequence. MFI is calculated as the average of the predominant flow in each of the four quadrants of the image and given punctuation between 0 and 3. Flow is characterized as absent flow (0), intermittent flow (1), sluggish flow (2) or normal constant flow (3).

Vascular density (TDV and PVD) evaluates the number of vessels in the examined site. Vascular density is calculated as the length of the vessels in relation to the total surface of the image (mm/mm^2^). Perfused vessels are those with a constant or sluggish flow, and non–perfused vessels are those with intermittent or absent flow. Vascular density was also calculated using the De Backer score by dividing the number of vessels that cross three equidistant horizontal parallel lines and three equidistant vertical parallel lines by the total length of the lines [22].

PPV% evaluates the functionality of the observed vessels. It refers to the percentage of perfused vessels of the total vessels in each sequence.

HI evaluates the heterogeneity or variability of predominant flow between the different sequences taken at a single time point at the different sites of evaluation. HI is calculated by measuring the difference between the highest MFI minus the lowest MFI divided by the mean flow velocity of all sublingual sites at a single time point.

All microcirculatory variables were calculated for small vessels (<20 μm) and for all vessels, according to the international consensus recommendations [21].

The cut-off point for the definition of an altered microcirculation was considered as an MFI for small vessels under 2.6 [10, 23–25].

### Statistical analysis

Data were analyzed using the IBM SPSS Statistics Version 20.0 (IBM, New York, NY) software. Central tendency measures (mean and median) and measures of dispersion (standard deviation and interquartile range) were used in the descriptive study. Mann–Whitney-Wilcoxon test was used to compare quantitative variables and the Fisher test to compare qualitative variables between the patients with and without evaluation of the microcirculation. The Pearson Correlation Coefficient (ρ) was used to analyze the correlation between microcirculatory parameters with other clinical parameters. Statistical significance was set at a *p* value of <0.05.

## Results

### Description of the sample

A total of 105 patients were included in the study, with a mean age of 4.6 ± 5.1 years (median 2.2 years) and a mean weight of 18.4 ± 16.6 kg (median 12.5 kg). Admission to the PICU was programmed in 45.7% of the patients, and 42.8% were after surgery. Table 1 reflects the reasons for admission. 25.3% of the patients were already intubated upon admission. Mean length of stay was 7.6 ± 10.5 days (median 4 days). 24 patients (22.9%) were discharged from the PICU in the first 24 h of admission.

**Table 1:** Reasons for admission to the Pediatric Intensive Care Unit

Cause of admission to the PICU	Total patients		Patients with evaluation of microcirculation (T_1_or T_2_)	
	n	%	n	%
Cardiac surgery	39	37.1	9	50
Respiratory disease	32	30.5	4	22.2
Cardiac failure	14	13.3	-	-
Neurosurgery	5	4.8	1	5.6
Cardiac arrest	3	2.9	3	16.7
Dehydration	3	2.9	-	-
Seizures	2	1.9	1	5.6
Diabetic ketoacidosis	1	1	-	-
Domiciliary ventilation control	1	1	-	-
Hypertensive crisis	1	1	-	-
Renal failure	1	1	-	-
Haemathemesis	1	1	-	-
Orthopaedic surgery	1	1	-	-
Septic shock	1	1	-	-
Total	105	100%	18	100%

### Evaluation of sublingual microcirculation

**Fig. 2:**
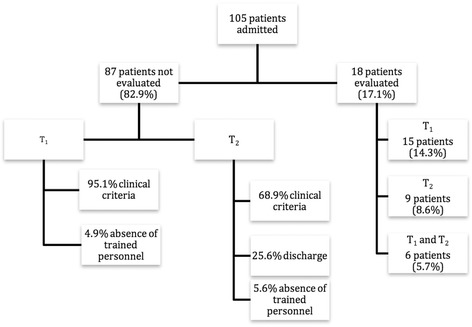
Distribution of the patients included in the study. T_1_: First 24 h of admission. T_2_: Second or third days of admission

Sublingual microcirculation was evaluated in 18 patients (17.1%) (Fig. 2). Sequences were taken in intubated patients in 95.2% of the cases. The reason for not evaluating sublingual microcirculation during the first day of admission (T_1_) was due to clinical criteria (lack of cooperation, presence of respiratory distress that may worsen with the procedure, etc.) in 95.1% of the cases and due to the lack of trained personnel in only 4.9% of the cases. Between the 2^nd^ and 3^rd^ days of admission (T_2_), microcirculation was not evaluated due to clinical criteria in 68.9% of the cases, lack of trained personnel in 5.6% and due to discharge from the PICU in 25.6% of the cases.

Table 2 compares the characteristics of patients in which microcirculation was evaluated and those in which it was not. Patients in which microcirculation was evaluated had a higher prevalence of intubation, ECMO, longer duration of mechanical ventilation, vasoactive drug therapy and length of PICU stay than the rest of the patients.

**Table 2:** Comparison between evaluated and non-evaluated patients

	Non evaluated patients (*n =* 87)		Patients with evaluation of microcirculation (*n =* 18)			All patients (*n =* 105)	
	n	%	n	%	p	n	%
Surgical	35	40.2%	10	55.6%	0.194	45	42.8%
Scheduled admission	38	43.7%	10	55.6%	0.439	48	45.7%
Intubated at admission	13	14.9%	13	72.2%	**<0.001**	26	24.7%
ECMO^*a*^	1	1.1%	3	16.7%	**0.015**	4	3.8%
Renal replacement therapy	7	8%	1	5.6%	1	8	7.6%
	**Median**	**IQR** ^***b***^	**Median**	**IQR** ^***b***^	**p**	**Median**	**IQR** ^***b***^
Age (years)	2.68	9	1.36	6.5	0.643	2.2	7.22
Weight (Kg)	13	22.2	12	12.9	0.589	12.5	16.3
Days on mechanical ventilation	0	0	6.5	18.25	**<0.001**	0	1
Days with vasoactive drugs	0	2	3.5	10.5	**0.014**	0	3
Length of stay (days)	3	6	13.5	16.5	**<0.001**	4	6.5

Statistically significant values marked in bold type

*ECMO* ^*a*^ Extracorporeal Membrane Oxygenation, *IQR* ^*b*^ InterQuartile Range

Table 3 shows microcirculatory parameters. Other clinical and analytic parameters are provided as Additional file 1: Table S1. Table 4 shows the values of microcirculation parameters at T_1_ and T_2_. There were no statistically significant differences in microcirculatory parameters between T_1_ and T_2_ (neither for small vessels nor for all vessels). There were no significant differences in microcirculatory parameters when comparing different diagnostic groups (data not shown).

**Table 3:** Microcirculatory parameters

	Small Vessels		All Vessels	
Microcirculatory parameters	Median	IQR^*f*^	Median	IQR^*f*^
MFI^*a*^	2.3	0.8	2.6	0.4
TVD^*b*^ (mm/mm2)	12.7	3.3	14.0	3.4
De Backer Score (vessels/mm)	8.9	1.8	-	-
PVD^*c*^ (mm/mm2)	12.8	3.2	13.3	4.0
PPV^*d*^ %	83.7	10.0	92.6	7.5
HI^*e*^	0.61	0.40	0.25	0.16

*MFI* ^*a*^ Microvascular Flow Index, *TVD* ^*b*^ Total Vessel Density, *PVD* ^*c*^ Perfused Vessel Density, *PPV* ^*d*^ Proportion of perfused vessels, *HI* ^*e*^ Heterogeneity Index, *IQR* ^*f*^ Interquartile range

**Table 4:** Microcirculatory parameters

	T_1_				T_2_			
	Small Vessels		All Vessels		Small Vessels		All Vessels	
	Median	IQR^*f*^	Median	IQR^*f*^	Median	IQR^*f*^	Median	IQR^*f*^
MFI^*a*^	2.1	0.9	2.5	0.4	2.4	0.6	2.7	0.3
TVD^*b*^ (mm/mm^2^)	12.4	4.1	13.3	3.5	12.7	2.3	14.7	3.5
DeBacker Score (vessels/mm)	8.6	1.9	NA^*g*^	NA^*g*^	9.4	1.5	NA^*g*^	NA^*g*^
PVD^*c*^ (mm/mm^2^)	12.0	3.5	12.5	4.3	13.0	2.8	13.3	3.1
PPV^*d*^ %	83.1	10.0	91.4	11.0	85.5	13.0	92.9	6.0
HI^*e*^	0.62	0.37	0.26	0.16	0.58	0.71	0.25	0.26

*MFI* ^*a*^ Microvascular Flow Index, *TVD* ^*b*^ Total Vessel Density, *PVD* ^*c*^ Perfused Vessel Density, *PPV* ^*d*^ Proportion of perfused vessels, *HI* ^*e*^ Heterogeneity Index, *IQR* ^*f*^ Interquartile range, *NA* ^*g*^ Non-Applicable

Only six patients had an evaluation of sublingual microcirculation at both T_1_ and T_2_ (Table 5), with no significant differences in any of the microcirculatory parameters between T_1_ and T_2_.

**Table 5:** Comparison between microcirculatory parameters at T1 and T2 in the 6 patients with both measurements

	Small Vessels					All Vessels				
	T1		T2			T1				p
	Median	IQR^*f*^	Median	IQR^*f*^	p	Median	IQR^*f*^	Median	IQR^*f*^	p
MFI^*a*^	2.1	1.4	2.3	1.1	1	2.6	0.9	2.7	-	0.157
TVD^*b*^ (mm/mm2)	14.6	3.6	12.7	1.9	0.317	16.0	2.8	14.7	-	0.157
De Backer Score (vessels/mm)	9.5	1.7	9.4	1.0	0.317	NA^*g*^	NA^*g*^	NA^*g*^	NA^*g*^	-
PVD^*c*^ (mm/mm2)	14.0	4.1	12.9	2.1	1	14.37	4.6	14.0	-	0.157
PPV^*d*^ %	84.2	21.5	81.3	14.1	1	92.65	19.9	95.7	-	0.157
HI^*e*^	0.63	0.70	0.50	0.95	0.317	0.23	0.38	0.22	-	1

*MFI* ^*a*^ Microvascular Flow Index, *TVD* ^*b*^ Total Vessel Density, *PVD* ^*c*^ Perfused Vessel Density, *PPV* ^*d*^ Proportion of perfused vessels, *HI* ^*e*^ Heterogeneity Index, *IQRf* Interquartile range, *NAg* Non-applicable

Microcirculation was altered in 70.8% of the measurements (defined as a MFI index for small vessels lower than 2.6). MFI was lower than 2.6 in 86.7% of the patients at T_1_ and in 55.6% of the patients at T_2_, but this difference did not reach statistical significance (*p =* 0.061).

### Correlation between microcirculatory parameters and other clinical variables

**Fig. 3:**
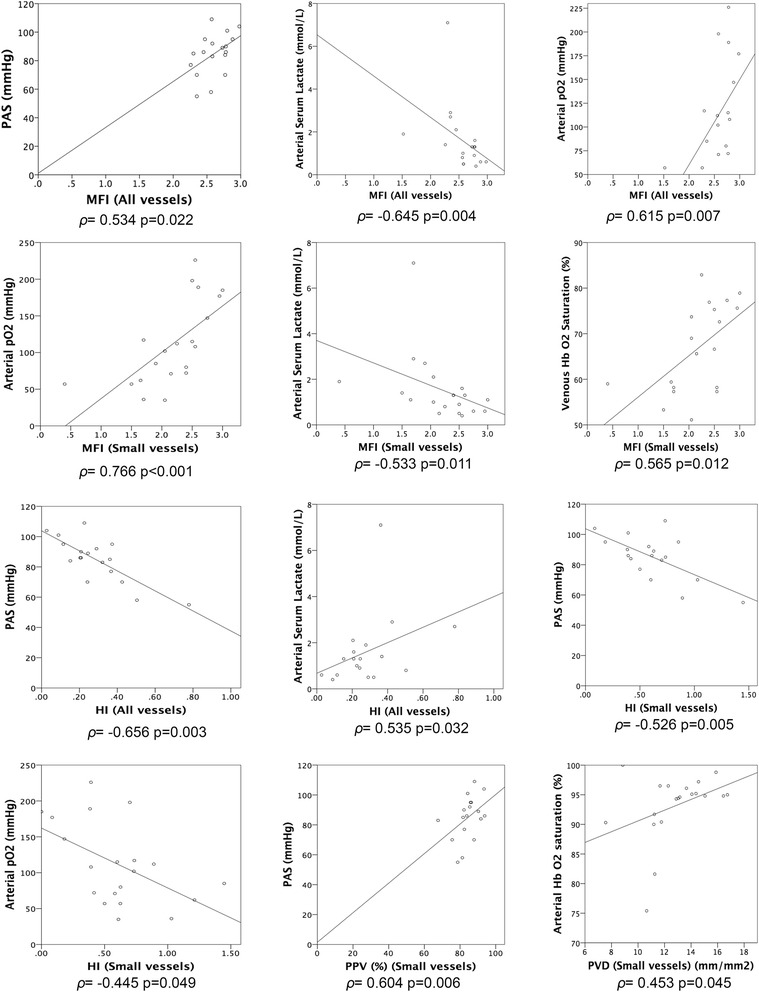
Scatterplot figures showing correlations found between microcirculatory parameters and other evaluated parameters. ρ: Spearman’s Rho. MFI: Microvascular Flow Index. HI: Heterogeneity Index. PPV(%): Proportion of perfused vessels. PVD: Perfused vessel density

Figure 3 shows the most important correlations between microcirculatory parameters and the rest of variables. There was a positive correlation between microcirculatory parameters and SAP, PaO_2_, temperature and central venous saturation. There was a negative correlation with CVP and lactate concentration.

### Prognostic value of microcirculatory parameters

Predictive power of microcirculatory parameters in the first 24 h of admission (T_1_) with respect to other clinical variables was studied. No correlation was found between microcirculatory parameters and PICU length of stay, mechanical ventilation, vasoactive drug therapy or ECMO. There were no differences in microcirculatory values between patients with a programmed versus an urgent admission to the PICU, or between patients in the postoperative period and non-surgical patients.

The small number of patients and the high prevalence of altered MFI made it impossible to make any comparisons regarding this parameter.

## Discussion

This is the first study evaluating the feasibility of routine microcirculatory evaluation with SDF microscopy in critically ill children.

Only a small percentage of patients (under 20%) admitted to the PICU were evaluated in our study. Very few evaluations were performed in non-intubated patients due to the lack of cooperation or to the presence of respiratory insufficiency. Cooperation from the patient, unless sedated and intubated, is one of the main limitations for the evaluation of microcirculation in children. To avoid this problem, some authors have proposed to assess microcirculation on the cheek instead of the oral mucosa in newborns and extubated patients [16, 19], making it less uncomfortable for the patient.

On the other hand, the device that is used for image acquisition is 210 mm long and weighs 425 gr, making it difficult to evaluate smaller children. A smaller device has been commercialized, which could be especially useful in smaller patients.

Despite all these limitations, microcirculation was assessed mostly in patients that were sicker, intubated and with longer duration of mechanical ventilation and vasoactive drug therapy. This is the kind of patient in which the study of microcirculation is more useful.

New technologies are usually designed for adults and adapting them to children is sometimes very difficult due to size discrepancies and lack of cooperation. Furthermore, when it comes down to critically ill children, determining the most adequate level of monitoring and invasiveness can be challenging, as more invasiveness usually involves a higher risk of complications. Developing a minimally invasive tool for real time evaluation of tissue delivery of oxygen and other nutrients in children would be a cornerstone in the management of critically ill patients. This would allow clinicians to directed management specifically to achieve an adequate tissue perfusion in very different pathologies. The design of a microcirculation evaluation device for children should have an adequate size according to the age of the patient and it should be easy to apply in order to minimize discomfort and, thus, rejection on behalf of the patient.

### Microcirculatory parameters and prevalence of microcirculatory alterations

Different microcirculatory parameters were evaluated. These parameters can be divided in three different groups: those evaluating vascular density (De Backer score, TVD and PVD), those evaluating the presence and characteristics of flow (PPV% and MFI) and those evaluating the variability between the types of predominant flow at different sites (HI).

Normal values of microcirculatory parameters, definition of microcirculatory alterations in critically ill patients and their relationship with prognosis have been described by different authors for adult patients [10, 26], but not for paediatric patients. An additional problem is that the characteristics of the microvascular compartment may change over time as the child grows [16]. Top et al. discovered that functional capillary density is higher in the neonatal period than in older children. Thus, studies describing characteristics, normal values and behaviour of the microvascular compartment in children are needed. There was considerable heterogeneity in microcirculatory parameters in our study population, but the limited number of patients and the high variability in clinical conditions make it impossible to stratify the analysis according to age.

MFI lower than 2.6 is considered the cut-off point to define an altered microcirculation in adult patients [23–25, 27]. The same cut-off point was used in our study, and the prevalence of altered microcirculation was much higher than what studies in critically ill adults show [10, 26]. More studies are needed to determine what the cut-off point should be used in children.

Regarding other parameters, functional vascular density was higher in our patients than what Top et al. describe in a different paediatric study [16]. This fact does not seem to be due to a worse microcirculatory situation since those patients did not have any respiratory or hemodynamic alterations. This difference is probably due to the use of a different device for image acquisition (OPS) and different software for image analysis. When different methods for evaluating a physiological phenomenon are used, results may not be comparable for some parameters. SDF and OPS have both been used to evaluate microcirculation. SDF was introduced after OPS and it has shown equivalent capacity to measure microcirculatory parameters providing higher image quality and a slightly higher image magnification. In the recent years, new devices have been developed using new imaging acquisition techniques (Incidental Dark Field Imaging and SDF +) with higher image resolution and real time image analysis [28]. The applicability and clinical utility of this new devices in the critically ill paediatric population has not been described yet.

### Correlation with other variables

Some macrohemodynamic parameters (SAP and CVP.) and some indicators of tissue perfusion (pH, venous oxygen saturation and lactate concentration) were related to some microcirculatory parameters in our study. This shows the deep interrelationship there is between macrocirculation, microcirculation and cell and tissue metabolism.

Since the number of patients in our study was small, it was not possible to analyse the relationship between microcirculatory alterations and the severity of the clinical condition, clinical evolution and the effect of different therapies. On the other hand, broader clinical and experimental studies are needed to assess how these compartments interact with each other.

Ideally, the use of microcirculation evaluation by direct imaging techniques should provide the clinician of a new tool to evaluate tissue perfusion. This new tool has been used in the adult population to guide treatment in different clinical conditions and to achieve an adequate tissue perfusion and oxygen delivery, showing advantages with regard to other commonly used indirect microcirculation evaluation techniques.

### Prognostic value of microcirculatory parameters

The analysis of the predictive capacity of microcirculation in terms of prognosis was not possible in our study due to the small number of patients in which microcirculation was evaluated and to the high prevalence of microcirculatory alterations. It is important to note that, according to the results of our study, paediatric patients in whom the evaluation of sublingual microcirculation is more feasible are those with a worse clinical condition. Thus, this technique should be considered in the assessment of critically ill patients that are at risk of having an altered tissue perfusion.

Multicentre studies including a larger number of patients and a greater percentage of less severe patients are needed in order to establish the prognostic capacity of microcirculatory alterations in children.

## Conclusions

The evaluation of sublingual microcirculation in critically ill children can be useful for the assessment of the microvascular compartment. It is minimally invasive and it is mainly indicated in sedated and ventilated patients.

Our results may serve as references for other paediatric studies. Nevertheless, more studies are needed to assess what microcirculatory values must be considered normal or pathological in children of different ages, to define the prevalence of microcirculatory alterations and their prognostic capacity in critically ill children.

## Additional file

Additional file 1: Table S1. Clinical and analytical parameters. (DOCX 78 kb)

## Abbreviations

CVP: Central venous pressure
DAP: Diastolic arterial pressure
ECMO: Extracorporeal membrane oxygenation
HI: Heterogeneity index
HR: Heart rate
MAP: Mean arterial pressure
MFI: Microvascular flow index
MV: Mechanical ventilation
NIRS: Near Infra-Red Spectrophotometry
OPS: Orthogonal polarization spectral
PICU: Paediatric intensive care
PPV%: Proportion of perfused vessels
PVD: Perfused vessel density
SAP: Systolic arterial pressure
SatO_2_%: Transcutaneous oxygen saturation
SDF: Sidestream dark field
TVD: Total vascular density
