# Supplementary material for: Evaluation of sublingual microcirculation in a paediatric intensive care unit: prospective observational study about its feasibility and utility
Source: BMC Pediatr. 2017 Mar 15;17:75. doi: 10.1186/s12887-017-0837-5 (PMC5353860; doi:10.1186/s12887-017-0837-5)
Supplement: Additional file 1: Table S1. — Clinical and analytical parameters. (DOCX 78 kb) [file 12887_2017_837_MOESM1_ESM.docx]

**Supplementary table: Clinical and analytical parameters.**

| **Clinical parameters** | **Median** | **IQR*^a^*** |
| --- | --- | --- |
| Heart Rate (bpm) | 125 | 16.3 |
| Systolic Arterial Pressure (mmHg) | 86 | 19.8 |
| Diastolic Arterial Pressure (mmHg) | 46 | 12.3 |
| Mean Arterial Pressure (mmHg) | 60 | 13.5 |
| Central Venous Pressure (mmHg) | 8.5 | 7 |
| Core Temperature (ºC) | 37 | 2.1 |
| Core to Peripheral Temperature difference (ºC) | 4 | 5.2 |
| Inotropic Score | 10.5 | 20.5 |
| **Analytical parameters** | **Median** | **IQR*^a^*** |
| Haemoglobin (g/dL) | 10.8 | 2.2 |
| Arterial pH | 7.40 | 0.10 |
| Arterial pCO_2_ (torr [kPa]) | 43 [5.73] | 9 [1.19] |
| Arterial PO_2_ (torr [kPa]) | 105 [14] | 105 [105] |
| Arterial lactate (mmol/L) | 1.2 | 1.2 |
| Venous pH | 7.34 | 0.09 |
| Venous pCO_2_ (torr [kPa]) | 51 [6.8] | 16 [2.13] |
| Venous pO_2_ (torr [kPa]) | 34 [4.53] | 13 [1.73] |
| Central Venous O_2_ Saturation (%) | 67 | 17 |
| O_2_ Extraction Ratio (%) | 29.0 | 20.8 |

IQR*^a^*: Interquartile range.
